# Totally intracorporeal colorectal anastomosis (TICA) versus classical mini-laparotomy for specimen extraction, after segmental bowel resection for deep endometriosis: a single-center experience

**DOI:** 10.1007/s00404-024-07412-6

**Published:** 2024-03-21

**Authors:** Manuel Maria Ianieri, Alessandra De Cicco Nardone, Pierfrancesco Greco, Antonella Carcagnì, Federica Campolo, Fabio Pacelli, Giovanni Scambia, Francesco Santullo

**Affiliations:** 1 Unit of Oncological Gynecology, Women’s Children’s and Public Health Department, Fondazione Policlinico Universitario Agostino Gemelli, IRCCS, Rome, Italy; 2 Gynecology and Breast Care Center, Mater Olbia Hospital, Olbia, Italy; 3 Catholic University of the Sacred Heart, Rome, Italy; 4 Epidemiology and Biostatistics Research Core Facility, Gemelli Generator, Fondazione Policlinico Universitario Agostino Gemelli IRCCS, Rome, Italy; 5 Surgical Unit of Peritoneum and Retroperitoneum, Fondazione Policlinico Universitario Agostino Gemelli IRCCS, Rome, Italy

**Keywords:** Bowel resection, Bowel endometriosis, Intracorporeal anastomosis, Deep endometriosis, Complications, Mini-laparotomy

## Abstract

**Purpose:**

The surgical approach to bowel endometriosis is still unclear. The aim of the study is to compare TICA to conventional specimen extractions and extra-abdominal insertion of the anvil in terms of both complications and functional outcomes.

**Methods:**

This is a single-center, observational, retrospective study conducted enrolling symptomatic women underwent laparoscopic excision of deep endometriosis with segmental bowel resection between September 2019 and June 2022. Women who underwent TICA were compared to classical technique (CT) in terms of intra- and postoperative complications, moreover, functional outcomes relating to the pelvic organs were assessed using validated questionnaires [Knowles-Eccersley-Scott-Symptom (KESS) questionnaire and Gastro-Intestinal Quality of Life Index (GIQLI)] for bowel function. Pain symptoms were assessed using Visual Analogue Scale (VAS) scores.

**Results:**

The sample included 64 women. TICA was performed on 31.2% (n = 20) of the women, whereas CT was used on 68.8% (n = 44). None of the patients experienced rectovaginal, vesicovaginal, ureteral or vesical fistula, or ureteral stenosis and uroperitoneum, and in no cases was it necessary to reoperate. Regarding the two surgical approaches, no significant difference was observed in terms of complications. As concerns pain symptoms at 6-month follow-up evaluations on stratified data, except for dysuria, all VAS scales reported showed significant reductions between median values, for both surgery interventions. As well, significant improvements were further observed in KESS scores and overall GIQLI. Only the GIQLI evaluation was significantly smaller in the TICA group compared to CT after the 6-month follow-up.

**Conclusions:**

We did not find any significant differences in terms of intra- or post-operative complications compared TICA and CT, but only a slight improvement in the Gastro-Intestinal Quality of Life Index in patients who underwent the CT compared to the TICA technique.

## What does this study add to the clinical work

**Table:** 

This study shows for the first time the functional outcomes and complications of a new surgical technique for intestinal resections for deep endometriosis with completely intracorporeal anastomosis and compares them with those of the classic technique.	

## Introduction

Deep endometriosis (DE), defined as endometrial glands and stroma infiltrating the peritoneum by at least 5 mm, is the most severe form of endometriosis [1]. Within the DE spectrum, bowel endometriosis has been estimated to affect between 5 and 12% of patients [2]. The rectum and sigmoid are involved in up to 90% of all intestinal lesions [3], and a laparoscopic or robotic resection of the affected part of the bowel may be required in cases of either occlusive symptoms or non-responsive medical pain patients [4–6].

In recent years there has been growing interest in the extraction of specimens via transnatural orifices [7–12], i.e. through a transvaginal or transrectal route, thus avoiding the abdominal incisions described in classic techniques (mini-laparotomy), which, though smaller than a laparotomy, can be associated with complications and suboptimal aesthetic results [7, 8].

Currently, even though there are no universal guidelines recommending which extraction and anastomosis technique should be preferred as an alternative to the classic one, a natural orifice specimen extraction (NOSE) is the most widely used [9–12]. In 2021, however, totally intracorporeal colorectal anastomosis (TICA) was described for the first time by our group [13]. It involves the execution of completely intra-abdominal colorectal anastomosis and the extraction of the specimen from the incision used for the 12 mm trocar, which is also used to insert the linear stapler, without a mini-laparotomy.

The aim of this study has been to investigate the impact of TICA, compared to conventional specimen extractions and extra-abdominal insertion of the anvil in terms of both complications and functional outcomes, in patients who underwent segmental bowel resection for colorectal endometriosis.

## Materials and methods

### Study protocol

This is a single-center, observational, retrospective study, which is reported in accordance with the “Strengthening the Reporting of Observational Studies in Epidemiology” (STROBE) guidelines and checklist [14]. We retrieved data sets on symptomatic women who underwent laparoscopic excision of DE with segmental bowel resection between September 2019 and June 2022 from the electronic databases and clinical records of the tertiary academic center for Endometriosis of the Fondazione Policlinico-Universitario Agostino Gemelli IRCCS in Rome (Italy). Patients were divided into two groups according to the surgical technique used for anastomosis and the extraction of specimens: either the classic technique (CT) or TICA. Pre-operative and post-operative functional outcomes, as well as differences between pre-operative and post-operative ones, were compared for the two groups.

### Ethics statement

This study received approval from the Institutional Review Board of the “Dipartimento Universitario Scienze della Vita e di Sanità Publica” (IRB protocol number DIPUSVSP-PD-07–234) and was carried out in accordance with the Helsinki Declaration. During pre-operative evaluation, patients were asked in advance to sign a consent form regarding the subsequent use of their anonymized data.

### Variables and procedures

Segmental bowel resection was performed in cases of patients for whom medical therapy had failed to control symptoms (i.e. progestins or estro-progestins), with simultaneous bowel obstruction or nodule residue > 3 cm after a shaving technique, or in cases of multiple bowel nodules. All the women had a histologically confirmed diagnosis of endometriosis. We excluded all women aged < 18 years, women who had previous discoid or segmental bowel resection for any benign or malignant diseases, or pelvic external beam radiotherapy/brachytherapy, or a concomitant diagnosis of diabetic microangiopathy/vasculopathies.

Retrieved data included medical and surgical history from the pre-operative evaluation. Moreover, all the women were subjected to recto-vaginal examination, dedicated transvaginal and transabdominal ultrasonography and/or pelvic magnetic resonance imaging. In cases of sub-occlusive symptoms, either a colonoscopy, a double barium enema or a virtual colonoscopy was also required to evaluate stenosis. Along with the above, interviews on pain symptoms and questionnaires on gastrointestinal function were also conducted.

Specifically, we focused on the main demographic, anthropometric and clinical data (i.e. age, body mass index, and previous surgery), clinical variables (pain and gastrointestinal symptoms), surgical findings (operating time, estimated blood loss, any intraoperative complications, length of resection, distance of the nodule from anal verge, and the need for ileostomy), and peri-operative data (days of hospitalization, need for self-catheterization, and post-operative complications).

Post-operative complications, occurring within 30 days after surgery, were described using the Clavien-Dindo classification [15]. Six months after surgery, patients underwent recto-vaginal evaluation and transvaginal and transabdominal ultrasonography. Interviews regarding pain symptoms and questionnaires were also reassessed (at the six-month follow-up visit). The severity of pain symptoms (dysmenorrhea, dysuria, dyschezia, and dyspareunia) was assessed using Visual Analogue Scale (VAS) scores (ranging from 0 to 10, i.e. from absence of pain to most severe).

Information regarding gastrointestinal functional outcomes was assessed using validated questionnaires: the Knowles-Eccersley-Scott-Symptom Questionnaire (KESS) [16] and the Gastro-Intestinal Quality of Life Index (GIQLI) [17]. The KESS questionnaire was used to assess bowel function and specifically determine whether the patient suffered from constipation (0 to 39 points). We used a cut-off criterion of ≥ 10 points in the total KESS score to define constipation [17]. The GIQLI was used to describe the health-related quality of life (QoL) of patients with gastrointestinal disease (0 to 144 points). The questionnaire consists of 36 items and a higher score indicates a better QoL [17]. Urinary retention was defined as a post-voiding residual volume of 100 mL. In these cases, self-catheterization was recommended until the post-urinary residual volume was < 100 mL at three consecutive measurements.

### Endpoints and outcome assessment

The primary endpoint of the study was to evaluate surgical outcomes, such as intra-operative, peri- and post-operative complications in women who underwent segmental bowel resection using the CT or the TICA technique. As secondary endpoints, we looked at gastrointestinal functional outcomes assessed using validated questionnaires, and pain symptoms both at baseline and at 6-month follow-up, to highlight potential improvements after intervention. Furthermore, we evaluated the correlation between functional outcomes at follow-up, by means of KESS and GIQLI questionnaires, and surgical, anthropometric and intra-operative findings.

#### Surgical technique and post-operative care

When preparing for surgery, all patients followed a 5-day residue-free diet and received mechanical bowel preparation in the form of a 4-L split dose of Macrogol: 2 L 2 days before surgery and 2 L the day before surgery. Intravenous cefuroxime and metronidazole were administered intraoperatively as antibiotic prophylaxis [13].

All patients were operated on by a multidisciplinary surgical team highly experienced in the laparoscopic surgical excision of bowel endometriosis, including a gynecologist and a colorectal surgeon. The severity of the disease was intra-operatively classified using the revised American-Fertility-Society (r-ASRM) score [18] and the *#*ENZIAN classification[19].

In all cases, a laparoscopic surgical approach for posterior DE using a nerve-sparing approach was used, as previously published [20–22]. When DE involved the lateral and/or posterior parametrium, a nerve-sparing parametrectomy was performed, as previously described by our group [1, 23]. In the case of further ureteral involvement due to the disease, ureterolysis was performed first and, if this failed to solve ureteral infiltration, ureteroneocistostomy was carried out [24, 25].

Segmental bowel resection was performed following the same steps, i.e. a 5 mm trocar was added in the right hypochondrium. Then the peritoneum of the mesosigma was opened above the root of the inferior mesenteric artery (IMA), as close to the bowel wall as possible. Sigmoid vessels, which supply the bowel segment to be resected, were progressively identified and selectively coagulated. The dissection was carried out until the rectal wall below the endometriotic nodule was reached, and then the rectum was transected with a linear stapler, the Echelon Flex™ Endopath® Stapler (EFES) 60 mm (Ethicon, Cincinnati, OH, USA). Colorectal anastomosis was performed by extracting the segment of bowel to be resected through a suprapubic mini-Pfannenstiel incision (4–5 cm) and following the classic steps [20, 26], or else with a totally intracorporeal anastomosis procedure (TICA) [27]. The choice of whether to use totally intracorporeal anastomosis (introduced at our institution in 2021) or a mini-Pfannenstiel incision was made at the discretion of the gynecologist and colorectal surgeon.

**Fig. 1:**
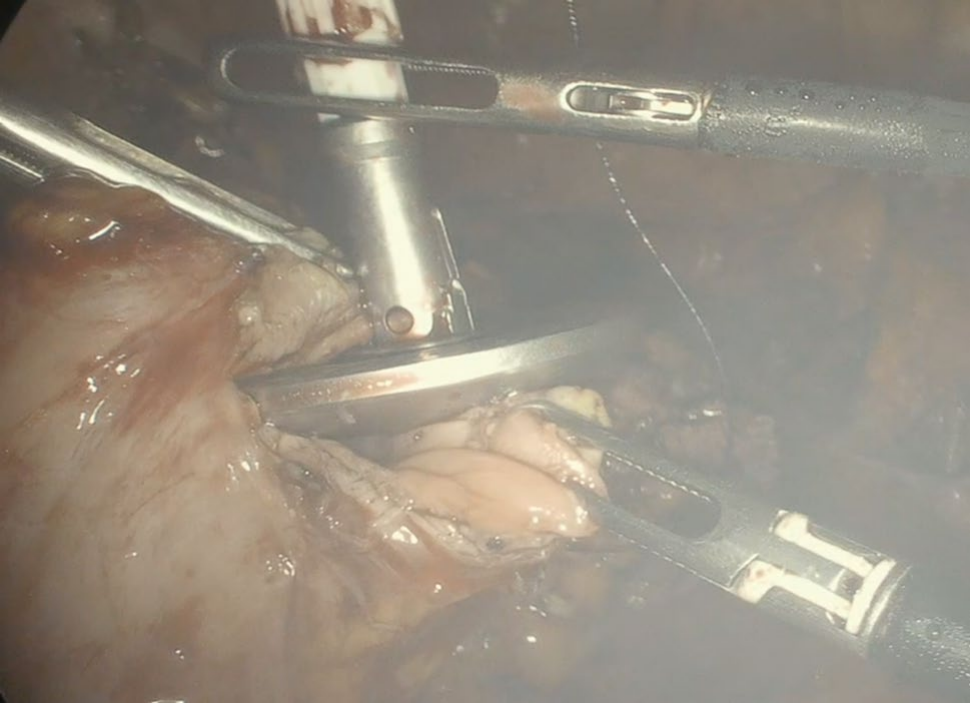
Anvil, prepared with a 0 vicryl suture, bound at the hole of the tip

**Fig. 2:**
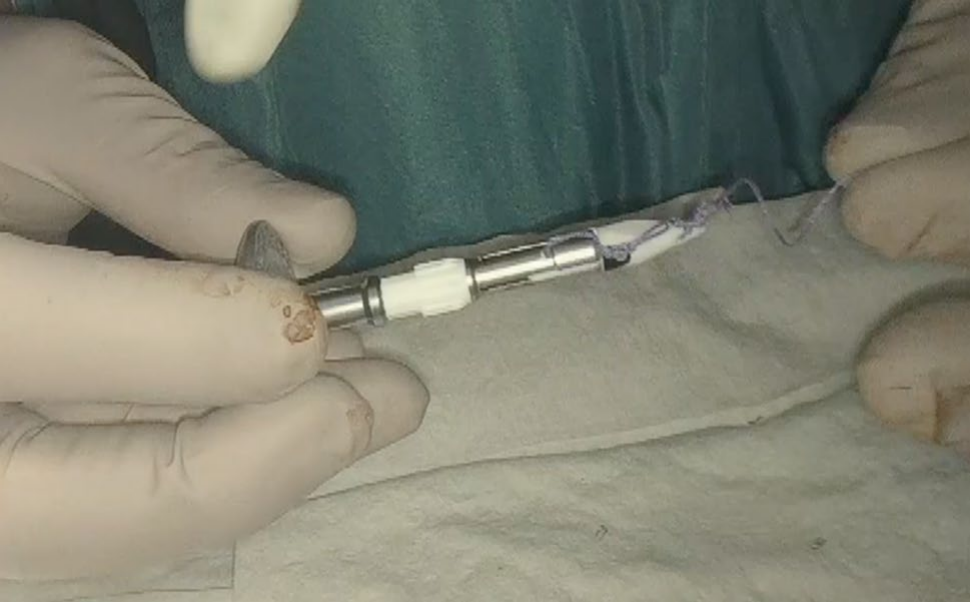
Anvil introduced through the colotomy perfomed cranially to the endometriotic nodule

**Fig. 3:**
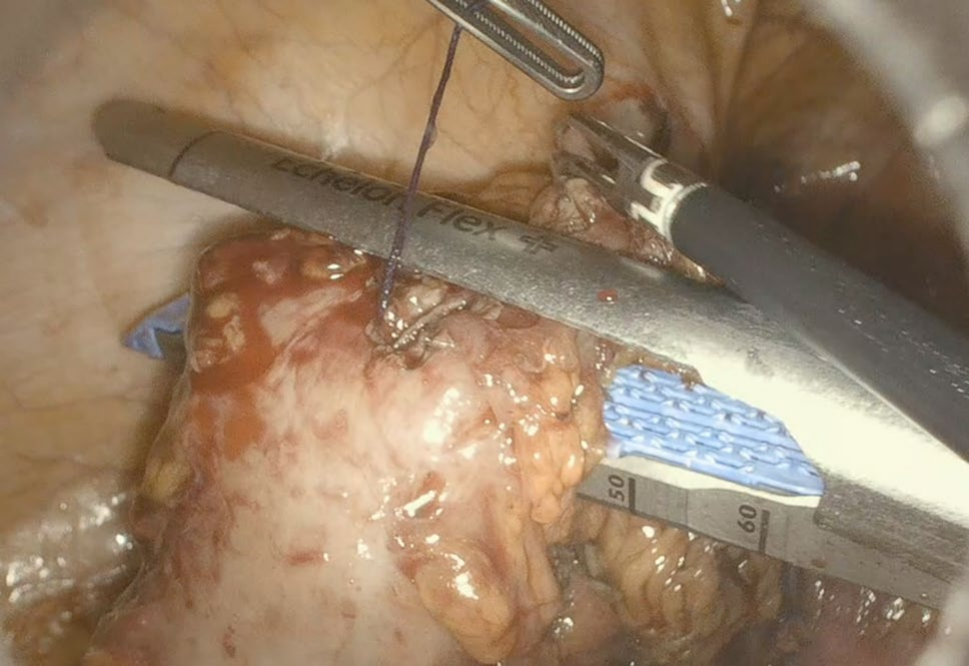
The stapler include the colotomy leaving the thread outside from the suture

**Fig. 4:**
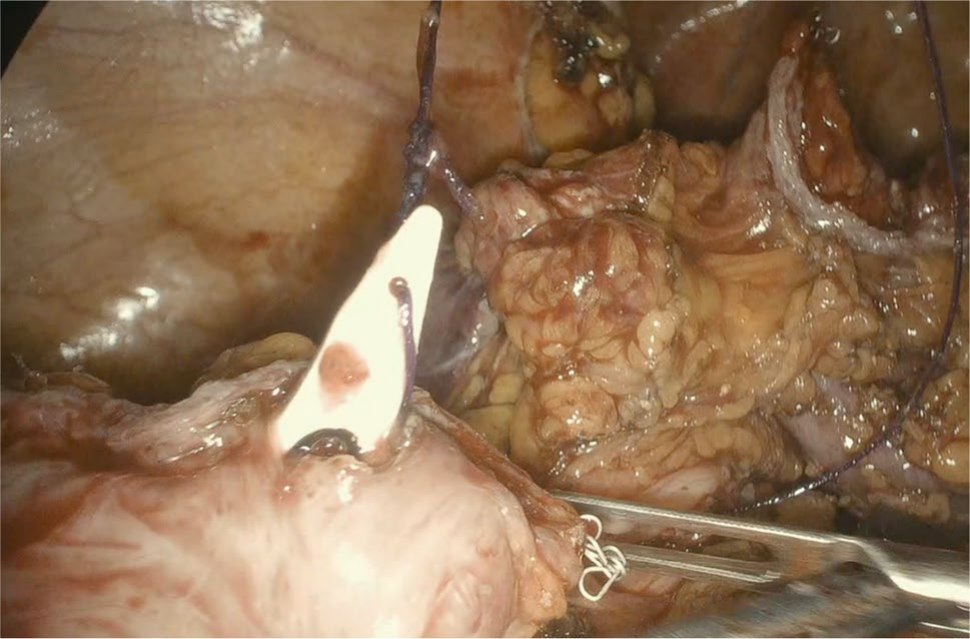
The anvil is extracted through the colon next to the suture line, pulling on the thread tied to it

Following the TICA technique, before anastomosis, the anvil of the circular stapling device (EEA™ circular stapler with Tri-Staple™ technology, 28 mm or 31 mm Medium/Thick, Covidien, New Haven, CT, USA) was prepared with a 0 vicryl suture, bound at the hole of the tip (Fig. 1). The anvil was brought into the abdominal cavity through the opening for the 12 mm port in the right abdominal flank. A colotomy was performed at the colonic wall just proximal to the endometriotic nodule, and then the anvil was introduced into the colon through the colotomy (Fig. 2). The linear stapler was arranged to include the whole colostomy. The suture attached to the rod of the anvil needed to be held from the superior edge of the colotomy, keeping the vicryl suture out of the linear stapler. The colon was then transected with a linear stapler (Fig. 3) and the anvil extracted through the colon next to the suture line, pulling on the thread tied to it (Fig. 4). Then the circular stapler was introduced in the rectum and end-to-end anastomosis was performed. The specimen was extracted through the 12 mm port on the right flank or through the vagina in cases of hysterectomy. In case of bowel segments with nodules too large to be extracted from a 12 mm incision, the specimen was partially morcellated with cold scissors in an endobag. At the end of the procedure, an air leak test was performed to evaluate anastomosis integrity. One drainage was left in place. In the post-operative period, at 3 and 5 post-operative days, a white cell count and C-reactive protein measurement were performed to look at potential early post-operative septic complications. Fast-track diet resumption was followed for nutrition.

### Statistical analysis

Given the retrospective observational nature of the study, it was not essential to resort to a formal determination of the sample size. But taking into account the number of patients who underwent surgery in the reference period and who strictly met the inclusion criteria, it was possible to enroll 64 patients.

The sample was described in its clinical and demographic characteristics using descriptive statistical techniques. Specifically, qualitative data sets were expressed as absolute and relative percentage frequencies, whereas quantitative variables as either mean and standard deviations (SD) or median and interquartile ranges (IQR), as appropriate. To verify the Gaussian distribution of quantitative variables, the Shapiro–Wilk test was applied.

Pre-post differences in the questionnaires on quality of life were analyzed using the Student’s t-test or the Wilcoxon rank-sum test for paired data, as appropriate. Finally, regression modeling was used to compare the pre-post differences between the two intervention techniques (i.e., estimated ∆-change differences), considering the CT as the reference point. Statistical significance was set at p-value < 0.05. All analyses were conducted using R software version 4.2.0 (CRAN ®, R Core 2022).

## Results

Table 1 shows the general characteristics of the study sample at baseline, overall and classified by surgery method (TICA vs CT). The sample included 64 women, with a mean age of 38.5 ± 6.1 years, and a median body mass index of 23.0 kg/m^2^ (IQR 20.4–24.6). TICA was performed on 31.2% (n = 20) of the women, whereas CT was used on 68.8% (n = 44). Over 32% (n = 21) of the women had previous surgery for endometriosis without bowel involvement; specifically, 35% (n = 7) in the TICA goup and over 31% (n = 14) in the CT group.

**Table 1:** General characteristics of the study sample at baseline (*N* = 64)

Variables	Overall (*n* = 64)	TICA (*n* = 20)	CT (*n* = 44)	*P*
Age (yrs)	38.5 (6.1)	38.0 (5.3)	38.8 (6.5)	0.629
BMI, kg/m^2^	23.0 (20.4–24.6)	21.1 (20–23.1)	23.5 (21–25)	0.064
Previous surgery for endometriosis	21 (32.8)	7 (35.0)	14 (31.8)	0.802
ASRM endometriosis stage				
I	2 (3.1)	0 (0.0)	2 (4.5)	0.305
II	2 (3.1)	1 (5.0)	1 (2.3)	
III	27 (42.2)	12 (60.0)	15 (34.1)	
IV	33 (51.6)	7 (35.0)	26 (59.1)	
VAS Pain Scales				
Dysuria	0 (0 – 2)	0 (0–3)	0 (0–3)	
Dysmenorrhea	8 (5–9)	8 (7–8)	8 (4–9)	
Dyspareunia	6 (3–7)	6 (5–7)	5 (1–7)	
Dyschezia	3 (0–7)	4 (1–7)	3 (0–7)	
Questionnaires				
KESS	19.2 (7.6)	18.4 (6.4)	19.3 (8.1)	0.638
GIQLI	57.3 (21.6)	50.8 (18.3)	60.2 (22.6)	0.083

BMI: Body mass index, ASRM: American Society for Reproductive Medicine, VAS: Visual Analog Scale, KESS: Knowles-Eccersley-Scott-Symptom Questionnaire, GIQLI: Gastro-Intestinal Quality of Life Index

Descriptive statistics are expressed as mean and standard deviations (SD) or medians (interquartile range: IQR) for quantitative variables, and as absolute and relative percentage frequencies for qualitative variables

Most of patients were classified as stage III and IV according to the American Society for Reproductive Medicine (ASRM) guidelines for endometriosis, with no significative differences between the two groups. Endometriosis was further mapped using the *#*Enzian Classification, as reported in Table 2.

**Table 2:** *#* Enzian Classification (N = 64)

*#* Enzian	Overall (n = 64)	TICA (n = 20)	CT (n = 44)	P
Peritoneum	18 (28.1)	3 (15.0)	15 (34.1)	0.202
Ovaries				
Absent	35 (54.7)	9 (45.0)	26 (59.1)	0.655
O1	12 (18.8)	4 (25.0)	8 (18.2)	
O2	14 (21.9)	6 (30.0)	8 (18.2)	
O3	3 (4.7)	1 (5.0)	2 (4.5)	
Tubes				
Absent	34 (53.1)	10 (50.0)	24 (54.5)	0.007
T1	7 (10.9)	4 (20.0)	3 (6.8)	
T2	12 (18.8)	0 (00.0)	12 (27.3)	
T3	11 (17.2)	6 (30.0)	11 (11.4)	
Compartments				
*A (rectovaginal septum and vagina)*				
Absent	34 (53.1)	11 (55.0)	23 (52.3)	0.691
A1	4 (6.2)	1 (5.0)	3 (6.8)	
A2	16 (25.0)	5 (25.0)	11 (25.0)	
A3	10 (15.6)	3 (15.0)	7 (15.9)	
*B (uterosacral/cardinal ligaments, parametrium, pelvic sidewalls)*				
Absent	24 (37.5)	7 (35.0)	17 (38.6)	0.801
B1	13 (20.3)	5 (25.0)	8 (18.2)	
B2	21 (32.8)	7 (35.0)	14 (31.8)	
B3	6 (9.4)	1 (5.0)	5 (11.4)	
*C (rectum)*				
Absent	4 (6.2)	0 (0.0)	4 (9.1)	0.180
C1	1 (1.6)	0 (0.0)	1 (2.3)	
C2	22 (34.4)	10 (50.0)	12 (27.3)	
C3	37 (57.8)	10 (50.0)	27 (61.4)	
Fa (adenomyosis)	46 (71.9)	14 (70.0)	32 (72.7)	0.822
Fb (urinary bladder involvement)	4 (6.2)	1 (5.0)	3 (6.8)	0.780
Fi (other intestinal locations)	4 (6.2)	0 (0)	4 (9.1)	
Fu (ureteric involvement with signs of obstruction)	8 (12.5)	2 (10.0)	6 (13.6)	0.683

Descriptive statistics are expressed as absolute and relative percentage frequencies

### Interventions

Table 3 shows the intervention data, both overall and classified by surgery method. The intestinal nodules were mainly single in both techniques, i.e. 80% n = 16 vs the 20% n = 4 that were multiple in the TICA group and 70.5% n = 31 vs the 29.5% n = 13 that were multiple in the CT group, with a mean size of 3.7 cm ± 1.0 (TICA) and 3.5 cm ± 0.7 (CT).

**Table 3:** Intervention data for the study population (*N* = 64)

Surgery	Overall (*n* = 64)	TICA (*n* = 20)	CT (*n* = 44)	P
Laparoscopy	64 (100)	20 (31.2)	44 (68.8)	
*Nodules*				
Single	47 (73.4)	16 (80.0)	31 (70.5)	0.422
Multiple	17 (26.6)	4 (20.0)	13 (29.5)	
Intestinal nodule size, cm	3.6 (0.8)	3.7 (1.0)	3.5 (0.7)	
Resected intestinal tract, cm	8 (6 – 9)	8 (6–9)	8 (6–9)	
*Intestinal tract*				
Rectum	35 (54.7)	13 (65.0)	22 (50.0)	0.762
Sigmoid	1 (1.6)	0 (0.0)	1 (2.3)	
Rectum + Sigmoid	24 (37.7)	7 (35.0)	17 (38.6)	
Rectum + Ileum	2 (3.1)	0 (0.0)	2 (4.5)	
Rectum + Ileocecal	2 (3.1)	0 (0.0)	2 (4.5)	
*Bowel anastomosis*				
L-L	5 (7.8)	0 (0.0)	5 (11.4)	0.011
L–T	10 (15.6)	0 (0.0)	10 (22.7)	
T-T	49 (76.6)	20 (100)	29 (65.9)	
Distance from the anal verge, cm	7 (6–8)	7 (6–7.3)	7 (6–9)	
Ileostomy	19 (30.0)	5 (25.0)	14 (31.8)	0.580
Colostomy	–	–	–	–
MSO	9 (14.1)	2 (10.0)	7 (15.9)	0.707
BSO	6 (9.4)	1 (5.0)	5 (11.4)	0.655
Ureterolysis	48 (75.0)	13 (65.0)	35 (79.5)	0.212
Neurolysis	8 (12.5)	3 (15.0)	5 (11.4)	0.683
Duration of intervention (minutes)	348 (77.9)	336.4 (77.7)	353.2 (76.7)	0.426
Days of hospitalization	6 (6–8)	6 (6–7.3)	6 (6–8)	0.597
Estimated blood loss, cc/mL	200 (150–300)	250 (187–300)	200 (150–300)	0.244
Other data				
Ureteral resection/reimplantation	3 (4.7)	1 (5.0)	2 (4.5)	0.936
Partial resection of the bladder	4 (6.2)	2 (10.0)	2 (10.0)	0.583
Partial vaginal resection	13 (20.3)	5 (25.0)	8 (18.2)	0.523
Conversion to laparotomy	–	–	–	–
Total hysterectomy	24 (37.5)	6 (30.0)	18 (40.9)	0.403
Posterolateral parametrectomy	51 (79.7)	18 (90.0)	33 (75.0)	0.166
Anterior parametrectomy	3 (4.7)	2 (10.0)	1 (2.3)	0.472

*TICA* totally intracorporeal anastomosis, *BSO* bilateral salpingo-oophorectomy,* MSO* monoliteral salpingo-oophorectomy,* T-T* termino-terminal,* L-T* latero-terminal,* L-L* latero-lateral

*Descriptive statistics are expressed as mean and standard deviations or median and interquartile ranges for quantitative variables, and as absolute and relative percentage frequencies for qualitative variables

In both the groups, the median resected intestinal tract was 8 cm (IQR 6–9) and the parts involved were mainly referred to the rectum (n = 13; 65% for TICA and n = 22; 50% for CT), though in 24 cases (n = 7 TICA, n = 17 CT) there was further association of the sigmoid.

Colostomy was not performed on any of the patients, whereas temporary ileostomy was needed in 25% (n = 5) of cases in TICA groups and in 31.8% (n = 14) of cases in CT group.

Only in a few cases was salpingo-oophorectomy (monolateral and bilateral) performed. Moreover, ureterolysis was performed in 79.5% (n = 35) of patients in the CT group and 65% (n = 13) patients in the TICA groups.

The mean operative time was 336.4 ± 77.7 and 353.2 ± 76.7 min for TICA and TC, respectively. A median of 6 days of hospitalization were required overall.

Moreover, during intervention the median Estimated Blood Loss (EBL) was 200 mL (IQR 150–300) in the CT group and 250 (IQR 187–300) in TICA group. Ureteral resection or reimplantation was needed in 1 (5.0%) and 2 (4.5%) cases in TICA and CT group, respectively.

It is notable that posterolateral parametrectomy was needed in 75% (n = 33) of cases of CT and 90% (n = 18) of the TICA group. The 30.0% (n = 6) TICA patients required a total hysterectomy comparate with the 40.9% (n = 18) patients in the CT group. Finally, as concerns associations between the surgery methods, only the bowel anastomosis provided a significant result (P = 0.011).

### Intra- and post-operative complications

Next, data on intra- and post-operative complications rates are shown in Table 4. All the women had surgery performed in laparoscopy and no conversion was required.

**Table 4:** Intra- and post-operative complications rates (*N* = 64)

Complications	Overall (*n* = 64)	TICA (*n* = 20)	CT (*n* = 44)	*P*	
Intraoperative complications	2 (3.1)	0 (0.0)	2 (4.5)	0.846	
Transfusion	3 (4.7)	1 (5.0)	2 (4.5)	0.936	
Fever	11 (17.2)	3 (15.0)	8 (18.2)	0.754	
Subcutaneous hematoma	1 (1.6)	0 (0.0)	1 (2.3)	0.687	
Pelvic abscess	3 (4.7)	1 (5.0)	2 (4.5)	0.936	
Uroperitoneum	–	–	–	–	
Hemoperitoneum	1(1.6)	0 (0.0)	1 (2.3)	0.496	
Urinary tract infections	8 (12.5)	3 (15.00)	5 (11.4)	0.683	
Bladder voiding deficit	5 (7.8)	2 (10.0)	3 (6.8)	0.660	
Intestinal anastomosis leakage	1 (1.6)	0 (0.0)	1 (5.0)	0.683	
Anastomosis stenosis	–	–	–		
Rectovaginal fistula	–	–	–		
Vesicovaginal fistula	–	–	–		
Ureteral fistula	–	–	–		
Ureteral stenosis	–	–	–		
Vesical fistula	–	–	–		
Reintervention	–	–	–		
ClavienDindo maximum grade	0 (0–2)	0 (0–1)	0 (0–1)	0.119	
Days of catheterization	4 (0–45)	22 (13–45)	2 (0–5)	0.603	
Time from surgery to flatus passage	2 (1–4)	2 (2—3)	2 (2–4)	0.113	

Descriptive statistics are expressed as median and interquartile ranges for quantitative variables, and as absolute and relative percentage frequencies for qualitative variables

The rate of intra-operative complications was extremely low (n = 2; 4.5% in CT vs n = 0 in TICA). Of note, none of the patients experienced rectovaginal, vesicovaginal, ureteral or vesical fistula, or ureteral stenosis and uroperitoneum. In one case (2.3%), hemoperitoneum was reported in the CT group but was treated conservatively. In one case (5.0%), intestinal anastomosis leakage was reported in the CT group, but the patient was underwent to protective ileostomy during the surgery, so she not required a reintervention.

No cases of reintervention were recorded, while bladder voiding deficit was observed in 10% of cases (n = 2) in the TICA group and 6.8% (n = 3) in the CT group. Urinary tract infections were observed in 15% (n = 3) in the TICA group and over 11% (n = 5) in the CT group.

Regarding the two surgical approaches, no significant difference was observed in terms of complications.

### Post-operative evaluation and questionnaires

Finally, as concerns pain symptoms at 6-month follow-up evaluations on stratified data, except for dysuria, all VAS scales reported showed significant reductions between median values, with an overall disappearance of symptom perception for both surgery interventions. As well, significant improvements were further observed in KESS scores and overall GIQLI. All these data sets are reported in Table 5.

**Table 5:** Pain VAS scale and questionnaire evaluations before intervention and at 6-month follow-up (N = 64)

	TICA			CT		
	Baseline	6-month FU	*p*	Baseline	6-month FU	*p*
VAS						
Dysuria	0 (0–3)	0 (0–0)	0.115	0 (0–0.3)	0 (0–0)	0.067
Dysmenorrhea	8 (7–8)	0 (0–0)	< 0.001	8 (3.8–9)	0 (0–0)	< 0.001
Dyspareunia	6 (5–7)	0 (0–2)	< 0.001	5 (0.7–7)	0 (0–1)	< 0.001
Dyschezia	4 (1–7)	0 (0–0)	< 0.001	3 (0–7)	0 (0–0)	< 0.001
Questionnaires						
KESS	18.4 (7.6)	12.1 (7.2)	0.002	19.3 (8.1)	13.0 (7.8)	< 0.001
GIQLI	50.8 (22.1)	73.8 (20.5)	0.003	60.2 (22.6)	90.5(18.2)	< 0.001

VAS: Visual Analog Scale, KESS: Knowles-Eccersley-Scott-Symptom Questionnaire, GIQLI: Gastro-Intestinal Quality of Life Index, FU: Follow-up

Descriptive statistics are expressed as mean and standard deviations or median and interquartile ranges

P-values were computed using either Student’s t test or the Wilcoxon rank-sum test for paired data

Table 6 shows the estimated ∆-change differences (pre-post) between the two intervention techniques for each outcome considered. Notably, only the GIQLI evaluation was significant after the 6-month follow-up (-14.119, P = 0.011).

**Table 6:** 6-month follow-up Δ-change differences* in the pain VAS scale and questionnaires

VAS (Ref.: CT)	6-month FU estimated Δ-change differences	P
Dysuria	− 0.960	0.621
Dysmenorrhea	3.481	0.339
Dyspareunia	0.855	0.483
Dyschezia	− 3.199	0.175

Questionnaires (Ref.: CT)	6-months FU estimated Δ-change differences	P
KESS	− 1.317	0.453
GIQLI	− 14.119	0.011

VAS: Visual Analog Scale; KESS: Knowles-Eccersley-Scott-Symptom Questionnaire; GIQLI: Gastro-Intestinal Quality of Life Index; FU: follow-up

^*^P-values were computed using regression modelling

Concerning the VAS score, dysuria and dyschezia revealed negative differences, in both questionnaire scores.

## Discussion

In our study, we compared two different segmental bowel resection techniques for endometriosis. We did not find any significant differences in terms of intra- or post-operative complications, but only a slight improvement in the Gastro-Intestinal Quality of Life Index in patients who underwent the CT compared to the TICA technique.

Other authors had previously demonstrated the feasibility and safety of the NOSE technique for bowel resection in DE using both the transvaginal and transrectal routes, for the extraction of the specimen [10, 28, 31]. In agreement with these studies, we also observed no statistically significant differences in terms of post-operative complications between the TICA technique and the classical one.

There were no major III-IV complications according to the Clavien-Dindo scale. Our data sets are comparable to the results reported by other authors ranging from 2.4 to 13.2% in terms of rectovaginal fistulas, vesicovaginal fistulas, anastomosis stenosis, ureteral fistulas, and bladder fistulas [8, 33]. The low rate of recto-vaginal fistulas and low post-operative complications is also supported by a recent study by Spagnolo et al., with 99 patients who, when comparing transvaginal specimen extraction (n = 23) and the classic technique (n = 76), showed no statistically significant differences in term of post-operative complications and recto-vaginal fistula rates between the groups [12]. Akladios et al., examining a group of 39 patients undergoing bowel resection for DE, observed a post-operative complication rate of 12.5% in those to whom the classic technique was applied and 20% in those who had the NOSE technique, with no statistically significant differences [29].

Moreover, these findings are also in agreement with two recent meta-analyses comparing the NOSE technique with the classic bowel resection technique for colorectal cancer; they showed that there were no substantial differences between the two techniques in terms of post-operative complications [8, 33]. These studies show that transvaginal and transanal NOSE techniques are as safe as the classic suprapubic technique when it comes to post-operative complications.

Our study, in particular, indicated that, in the TICA group, leakage of the anastomosis never occurred, in contrast to the CT group, where it occurred in only one case (5%) (patient who had already undergone surgery for DE). The leakage rate is essentially the one indicated in the literature, which ranges between 0 and 3% [10, 28, 31, 33, 34]. Obviously, given the small number of patients, we cannot determine definitively whether the TICA technique is safer in terms of leakage compared to CT (p = 0.683).

It is interesting to observe that one of the most frequent post-operative complications for both techniques was bladder voiding deficit (10%, 2 patients for TICA vs 6.8%, 3 patients for CT). The complication was resolved with the use of intermittent self-catheterization within 45 days from surgery in each of the 5 patients. However, this rate was lower than in other studies on nerve-sparing techniques (0–22%) [35]. Nonetheless, this comparison is not reliable, as only a few studies have specified parametrectomy, which is itself considered a risk factor for post-operative urinary retensionism [1, 20].

We did not observe any cases of reintervention, as the only anastomotic leak occurred in a patient for whom a temporary ileostomy had previously been performed due to the low distance of anastomosis from the anal margin. As such, the patient was treated conservatively, maintaining the stoma for 70 days, and it then closed without complications; performing a barium enema confirmed the healing of the millimetric colorectal dehiscence.

Bowel function, on the other hand, improved significantly in our series, as confirmed by the considerable enhancement of both KESS (P < 0.001) and GIQLI (P < 0.001) after colorectal surgery. Conversely, an other study have not shown any relief from digestive complaints after segmental bowel resection for DE [36]. Specifically, our study showed that the change in GIQLI score, between baseline and follow-up, in the TICA group was smaller (p = 0.011) than the same item in CT. This difference is probably due to the fact that the baseline score was slightly better in the CT group than in the TICA group.

What in our opinion most differentiates the two surgical techniques is essentially the number of staples necessary for the resection. In fact, in the TICA technique, compared to a potential advantage in reducing laparotomy incisions, the use of an additional stapler is required to resect the cranial portion of the bowel segment with DE and fix the anvil, which instead in the CT is usually inserted manually into the intestinal lumen and blocked with a tobacco pouch. This additional suture on the bowel could theoretically represent an additional risk as in the NOSE, but in our series the only dehiscence was actually reported using the CT.

We know that our study’s most significant limitation is its retrospective nature and the relatively small sample, but the two groups are comparable from the point of view of clinical characteristics and intra-operative findings, which could reduce the initial bias. Nonetheless, our study is the first to compare the TICA technique with the classic one for segmental bowel resection for DE. Therefore we can argue that the TICA technique is as safe and feasible as the CT in terms of post-operative complications, and this technique can thus be considered an alternative to the NOSE, especially when opening the vagina is not planned.

## Data Availability

The data that support the findings of this study are available from the corresponding author, upon reasonable request.
